# New classification criteria for systemic lupus erythematosus correlate with disease activity

**DOI:** 10.3325/cmj.2014.55.514

**Published:** 2014-10

**Authors:** Felina Anić, Marta Žuvić-Butorac, Davor Štimac, Srđan Novak

**Affiliations:** 1 Department of Rheumatology and Clinical Immunology, Division of Internal Medicine, University Hospital Center Rijeka, Rijeka, Croatia; 2 Department of Biotechnology, University of Rijeka, Rijeka, Croatia; 3 Department of Gastroenterology, Division of Internal Medicine, University Hospital Center Rijeka, Rijeka, Croatia

## Abstract

**Aim:**

To determine the prevalence of American College of Rheumatology (ACR) and Systemic Lupus International Collaborating Clinics (SLICC) classification criteria among systemic lupus erythematosus (SLE) patients; to determine disease activity and severity; and to investigate the correlation of classification criteria with disease activity, and of disease activity and damage index with disease duration.

**Methods:**

We performed a cross-sectional study on 110 SLE patients from the Division of Rheumatology and Clinical Immunology, University Hospital Centre Rijeka, Croatia in the period from September to December 2013 and determined disease duration and the total number of ACR and SLICC classification criteria. Disease activity was assessed by Systemic Lupus Erythematosus Disease Activity Index (SLEDAI) index and organ damage by Systemic Lupus International Collaborating Clinics/American College of Rheumatology (SLICC/ACR) damage index.

**Results:**

The number of SLICC classification criteria met per patient was significantly higher than the number of ACR criteria (7 [IQR 6-8] vs 5 [IQR 4-6], *P* < 0.001). Moderate correlations were detected between the number of SLICC classification criteria and disease activity index, both in case of active (r = 0.48, *P* = 0.003) and inactive disease (r = 0.43, *P* < 0.001). We neither found a correlation between the number of ACR criteria and disease activity nor between disease activity and disease duration. However, there was a good correlation between SLICC/ACR damage index and disease duration (r = 0.63, *P* < 0.001).

**Conclusion:**

New SLICC classification criteria correlate with disease activity because they capture more manifestations also included in the SLEDAI index. Patients with longer disease duration had a larger damage index score.

Systemic lupus erythematosus (SLE) is a chronic inflammatory disease affecting a number of organs and organ systems (1,2). The first classification criteria for SLE were developed in 1971, revised in 1982 (3), and adopted by the American College of Rheumatology (ACR) in 1997 (1). These criteria were developed and validated for the classification of patients with a longstanding established disease. Although developed as “classification criteria,” ACR criteria have been extensively used as diagnostic criteria. For diagnosis of SLE, the patient must satisfy at least 4 of 11 ACR classification criteria. These criteria were revised and validated by the Systemic Lupus International Collaborating Clinics (SLICC) group in 2012 (4), and according to SLICC, the patient must satisfy at least 4 of 17 SLICC classification criteria, including at least one clinical and one immunologic criterion. In Croatia only two studies so far have determined the prevalence of ACR classification criteria among patients with SLE (5,6).

The Systemic Lupus Erythematosus Disease Activity Index (SLEDAI) is one of the standard scales utilized to assess disease activity (7-9). A few modifications of SLEDAI index have been made (Mex-SLEDAI, SLEDAI-2K, SELENA SLEDAI), one of them in the Safety of Estrogens in Lupus Erythematosus National Assessment (SELENA) trial known as the SELENA-SLEDAI system (10). Despite the modifications of some of the descriptors, SELENA SLEDAI is very similar to SLEDAI-2K. The maximum possible score of SELENA SLEDAI index is 105.

The Systemic Lupus International Collaborating Clinics (SLICC/ACR) damage index has been developed to assess irreversible damage in SLE patients, independently of its cause (7,11,12). The maximum possible score is 47. The SLICC damage score gradually increases over time (13,14) and patients with higher damage scores early in the course of disease have been associated with poor prognosis and increased mortality (15,16).

SLICC classification criteria improved the clinical relevance of the ACR criteria, incorporated recent findings on the immunology of SLE, and resolved several problems attributed to the ACR criteria (4). As SLICC classification criteria have an impact on clinical practice, we wanted to see if they correlated with disease activity. It is known that disease duration in SLE patients affects organ damage (13,14), but data on the influence of disease duration on disease activity are missing. The aim of this study was 1) to determine the prevalence of each of the ACR and SLICC classification criteria and make a comparison between them, 2) to determine the correlation between both classification criteria and disease activity and 3) to determine the correlation between disease activity and damage index with disease duration.

## Patients and methods

### Patients

We performed a cross-sectional analysis of SLE patients from the Division of Rheumatology and Clinical Immunology, University Hospital Centre Rijeka, Croatia who fulfilled at least 4 ACR classification criteria and were examined by Division’s specialists from September to December 2013. Patients with fewer than 4 ACR classification criteria were not included. The patients either had a previously established diagnosis of SLE or were diagnosed at the last visit. Time limit of the study was set to 3 months because common outpatient examination period lasts for an average of 3 months. The study included all 110 consecutive patients with SLE who were examined by physicians at our hospital center during the period of 3 months and their medical records were analyzed. Among patients not included in the study, but examined in this period, 9 patients fulfilled ≥4 SLICC classification criteria, but did not fulfill 4 ACR classification criteria. All our patients included in the study fulfilled at least 4 ACR classification criteria while the number of fulfilled SLICC criteria was not an inclusion criterion. Median age of all patients was 47 years (range 20-75). There were 97 (88%) female and 13 (12%) male patients. Median age of all patients at diagnosis was 37 years (range 11-74). Median of SLE duration was 9 years (range 5-13).

### Methods

For each patient the cumulative and individual frequency of each of the ACR and SLICC classification criteria, SELENA SLEDAI components, and SLICC/ACR damage items were determined. ACR classification criteria revised 1982 with reference to their 1997 updated version and new SLICC classification criteria were used (1,3,4). ACR and SLICC classification criteria present at the last visit were taken into consideration, as well as all the criteria from the time when SLE diagnosis was established. All the criteria from the time when SLE diagnosis was made were captured. Disease activity was assessed using SELENA SLEDAI score-weighted scale for 24 parameters. SLE patients with SLEDAI score ≥6 were considered to have active disease. Damage was assessed by SLICC/ACR damage index. It is defined for 12 organ systems and had to be continuously present for at least 6 months. Damage score can only remain stable over time or increase, to a maximum of 47 points (11).

Data were analyzed using the STATISTICA software, version 12.0 (StatSoft, Inc., Tulsa, OK, USA). The normality of distribution was tested by Kolmogorov-Smirnov test. Non-normally distributed variables are shown as medians with interquartile range (IR). Nominal variables are presented as frequencies or percentages. For non-normally distributed values we used Mann-Whitney U-test and for normally distributed variables, Spearman rank correlation coefficient r and r^2^ value. Statistical significance level was *P* < 0.05. Correlations from 0 to 0.25 (or -0.25) were interpreted as no relation, those from 0.25 to 0.50 (or -0.25 to -0.50) as a fair degree of relation, those from 0.50 to 0.75 as a moderate to good relation, and those greater than 0.75 (or -0.75) as a very good to excellent relation.

## Results

**Figure 1:**
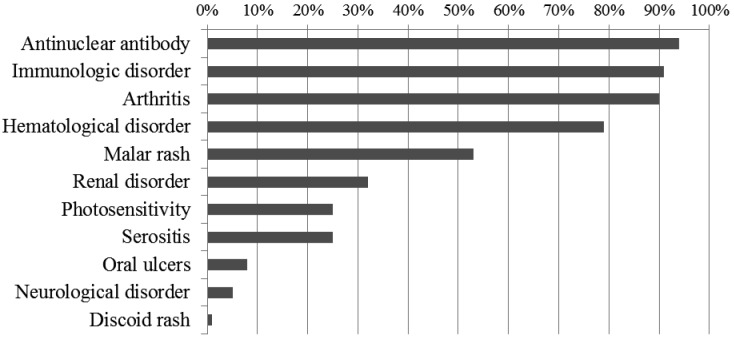
The prevalence of the American College of Rheumatology (ACR) classification criteria for systemic lupus erythematosus (SLE) in 110 SLE patients.

**Figure 2:**
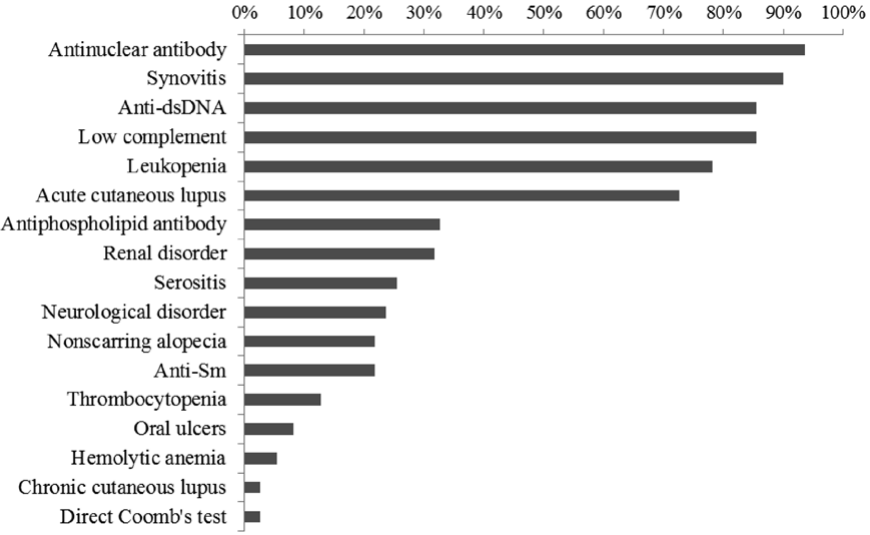
The prevalence of Systemic Lupus International Collaborating Clinics (SLICC) classification criteria for systemic lupus erythematosus (SLE) in 110 SLE patients.

**Figure 3:**
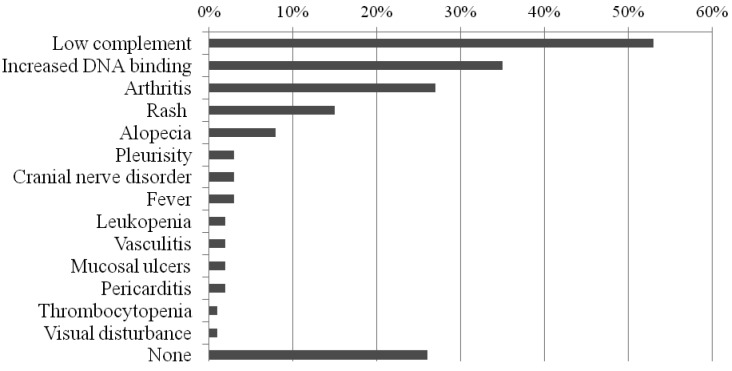
The prevalence of Systemic Lupus Erythematosus Disease Activity Index (SLEDAI) components in 110 SLE patients.

**Figure 4:**
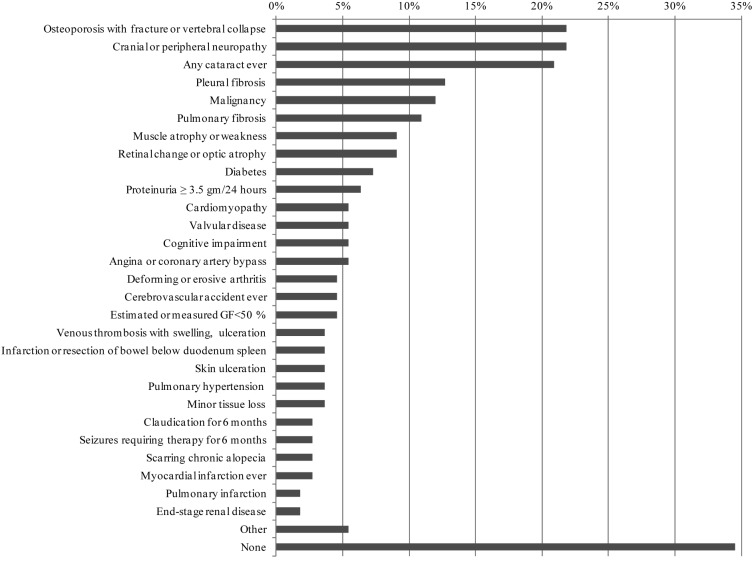
The prevalence of Systemic Lupus International Collaborating Clinics/American College of Rheumatology (SLICC/ACR) damage index components in 110 systemic lupus erythematosus (SLE) patients. “Other” include premature gonadal failure, pericarditis for 6 months or pericardiectomy, transverse myelitis, avascular necrosis, osteomyelitis, stricture of upper gastrointestinal tract surgery ever. GF – glomerular filtration.

The prevalence of each ACR classification criterion (Figure 1) and new SLICC criterion (Figure 2) for SLE was determined and the most frequently observed criteria were positive ANA titer (in 94% of patients), immunologic disorder (91%), arthritis (90%), anti-dsDNA (85%), low complement (85%), hematologic disorder (79%), leukopenia (78%), and acute cutaneous lupus (73%). For 11 (10%) SLE patients, there were no data about anticardiolipin antibodies. Median number of ACR classification criteria met per patient was 5 (IQR 4-6) and of SLICC classification criteria was 7 (IQR 6-8) (Mann-Whitney U test, *P* < 0.001). Thirty-six patients (33%) had active SLE (SELENA SLEDAI score ≥6). Median SELENA SLEDAI score of all patients was 2 (IQR 0-7), while median SELENA SLEDAI score of patients with active SLE was 8 (IQR 7-10). We found no correlation between ACR classification criteria and SELENA SLEDAI score either in active (r = 0.23, *P* = 0.173) or inactive (r = 0.24, *P* = 0.041) disease. However, moderate correlations were detected between SLICC classification criteria and disease activity index, both in active (r = 0.48, *P* = 0.003) and inactive disease (r = 0.43, *P* < 0.001) (Table 1). The most frequently observed clinical and laboratory components of SELENA SLEDAI index were low complement in 53%, increased DNA binding in 35%, arthritis in 27%, and rash in 15% of patients (Figure 3). Median SLICC/ACR damage index score of all patients was 2 (IQR 0-3). The most frequently observed components were osteoporosis with fracture or vertebral collapse and cranial or peripheral neuropathy in 22%, any cataract ever in 21%, pleural fibrosis in 13%, and malignant diseases in 12% of patients (Figure 4). Among SLE patients with malignant diseases we recorded 4 gynecologic cancers, 2 breast cancers, 1 melanoma, 1 basalioma, 1 kidney cancer, 1 lung cancer, 1 myeloproliferative neoplasm, 1 Hürthle cell cancer, and 1 metastatic brain tumor. This group had significantly longer disease duration than patients without malignant diseases (median 15 years [IQR 9-20] vs 9 years [IQR 5-13], *P* = 0.042). The correlation between activity score index and duration of disease was not found (r = -0.13, *P* = 0.172), although a good correlation between disease duration and SLICC/ACR damage index was recorded (r = 0.63, *P* < 0.001).

**Table 1:** Spearman rank correlation coefficients r and r^2^, and their respective level of statistical significance for association between the number of ACR classification criteria, SLICC classification criteria, SLICC/ACR damage index, and SELENA SLEDAI score in groups with active (n = 36) and inactive (n = 74) SLE

	SELENA SLEDAI score					
	Active SLE			Inactive SLE		
	r	r^2^	*P*	r	r^2^	*P*
ACR number of CC	0.23	0.05	0.173	0.24	0.06	0.041
SLICC number of CC	0.48	0.23	0.003	0.43	0.18	<0.001
SLICC/ACR damage index	0.18	0.03	0.291	0.01	0.00	0.817

*Abbreviations: ACR – American College of Rheumatology; CC – classification criteria; SLICC – Systemic Lupus International Collaborating Clinics; SLICC/ACR – Systemic Lupus International Collaborating Clinics/American College of Rheumatology; SELENA SLEDAI – Safety of Estrogens in Lupus Erythematosus National Assessment Systemic Lupus Erythematosus Disease Activity Index; SLE – systemic lupus erythematosus.

## Discussion

Our study found a moderate correlation between the number of new SLICC classification criteria and SLEDAI disease activity index, both in active and inactive disease. However, there was no correlation between the number of ACR classification criteria and disease activity index. SLICC classification criteria correlate with disease activity because they capture more clinical and laboratory findings also involved in SLEDAI index. In comparison to ACR criteria, SLICC criteria and SLEDAI index capture more neurological manifestations of disease (4,7). SLICC neurological disorder was observed in 24% of our patients, while ACR neurological disorder in only 5%. SLICC criteria and SLEDAI index also include nonscarring alopecia, which is not included in ACR criteria (4,7). Nonscarring alopecia was found in 22% of our patients. In comparison to ACR criteria, SLICC criteria and SLEDAI index include separately leucopenia and thrombocytopenia, which are important laboratory parameters of disease activity (4,7). In our study, ACR hematologic disorder was detected in 79%, leucopenia as a separate SLICC criterion in 78%, and trombocytopenia in 13% of patients. Low complement, found in a large number of our patients (85%), is not included in ACR criteria, but is in SLICC criteria (4). As a laboratory characteristic of disease activity, it contributes to correlation between new SLICC criteria and activity score index.

The prevalence of the ACR classification criteria in our patients was similar to that in previous studies (3,5,17-19). In comparison to other European studies, we detected more arthritis (90%) but less discoid rash (1%) and photosensitivity (25%).

Our study showed that patients with longer disease duration had a larger damage index score. In general, damage score remains the same over time or increases (20). Gladman et al have shown a gradual increase in damage score over a period of 15 years (13), while another study showed an increase over a period of 5 years in an average 30% of patients (20). In our study the most frequently observed feature of SLICC/ACR damage index was osteoporosis with fracture or vertebral collapse. It has been shown that the frequency of fractures in women with SLE is between 5.0 and 21.4% (21,22). Our analysis detected malignancy as the fifth most represented component of SLICC/ACR damage index. Another study found a greater number of cancer cases in SLE patients (10%-15%) than in the general population, due to the combination of baseline immune system defects and exposure to immunosuppressive medications (23). Increased risk of hematologic cancers and decreased risk of hormone-sensitive cancers caused by alterations in estrogen metabolism was reported in several studies (23,24). In contrast to this, we detected hormone-sensitive cancers in 6 SLE patients, while hematologic cancer was found in only one patient.

Our study has several limitations. It was conducted in only one center and we did not analyze if patients with active SLE also developed a higher damage score index. Data about performance of the new SLICC classification criteria in childhood SLE have been recently published (25). Our study showed that new SLICC classification criteria correlated with disease activity because they included more manifestations of SLE also involved in SLEDAI activity index. Although they were developed as classification criteria, SLICC criteria are more consistent and have substantial impact on clinical practice and probably in the future they will be used as diagnostic criteria. However, more research on a larger number of patients is needed in order to obtain more representative results.
